# Thermal Behaviour of Beams with Slant End-Plate Connection Subjected to Nonsymmetric Gravity Load

**DOI:** 10.1155/2014/323206

**Published:** 2014-01-23

**Authors:** Farshad Zahmatkesh, Mohd Hanim Osman, Elnaz Talebi

**Affiliations:** Department of Structures and Materials, Faculty of Civil Engineering, Universiti Teknologi Malaysia, 81310 Johor, Malaysia

## Abstract

Research on the steel structures with confining of axial expansion in fixed beams has been quite intensive in the past decade. It is well established that the thermal behaviour has a key influence on steel structural behaviours. This paper describes mechanical behaviour of beams with bolted slant end-plate connection with nonsymmetric gravity load, subjected to temperature increase. Furthermore, the performance of slant connections of beams in steel moment frame structures in the elastic field is investigated. The proposed model proved that this flexible connection system could successfully decrease the extra thermal induced axial force by both of the friction force dissipation among two faces of slant connection and a small upward movement on the slant plane. The applicability of primary assumption is illustrated. The results from the proposed model are examined within various slant angles, thermal and friction factors. It can be concluded that higher thermal conditions are tolerable when slanting connection is used.

## 1. Introduction

The most specified weakness of steel structures is reduction in its compressive strength during temperature increase and/or fire. Lack of strength in the steel elements mainly depend upon boundary conditions of the beams and columns in the end connections at the supports [1, 2]. The connections of a steel structure play a key role in controlling and carrying of initial axial forces and also gravity loads. Therefore, monitoring of thermal force at joints can be useful for probably structural failure.

The evaluation of the end connections at elevated temperature was a topic of many research programs in recent years. Liu et al. [1] has investigated the failure elevated temperature of the steel beams with axial restraints at the supports. The critical elevated temperature conditions of steel compressive members have also been studied by Rodrigues et al. [3]. Besides, the reaction of initial axial compressive force caused by the elevated temperature may lead to buckling of beam-columns. This is widely investigated by [4]. The results of these experimental tests confirmed that the restrained beam generate huge axial compressive force in the beam when it is subjected to elevated temperature [2, 3]. It is observed that the supports at two ends of the beam tend to resist against member expansion. Besides, the behaviour of beam to column joints at elevated temperature has been simulated by Al-Jabri [5] and also end connection behaviour has been tested experimentally by Qian et al. [2].

In steel structures, designers need to find an appropriate solution against thermal effects in fully axially restrained beams. Some of the most common solutions are (i) increasing section area, (ii) using from lateral supports, (iii) cooling system by air-conditioning, (iv) covering system by concrete or isolation, and (v) thermal break. Although it is important to select a suitable option to present more economically structures, most of the presented methods are extensively costly.

From the literature review, it can be seen that the bolted slant end-plate connections could be one of the most economically generated methods [6]. These connections can sustain against the large axial load subjected to temperature increase. The resulted equations of analytical model [6] show that, after an increase in temperature in the beams with conventional (vertical) connections, a huge extra axial force will be induced into the beam. This thermal axial force can decrease the capability of member to carry external symmetric-gravity loads. Thermal damping ability of connection can increase when details of it change to slanting type one. So, this type of connections can reduce induced thermal axial force and raise the capability of member against elevated temperature. In this paper, an analytical method is presented to derive equilibrium equations for a beam with nonsymmetric gravity load. The beam has been studied to find axial force and movement elevated temperature. From the analytical study, it can be found that the end-plate connection with slanting joint can be used as a damping device for the axial force induced in the beam due to elevated temperature.

From the literature review, it can be seen that the numerical and analytical approaches are very popular in the investigation of steel structures under elevated temperature. The effects of axial restraint have been investigated numerically by Shepherd and Burgess [7] and analytically by Wong [8]. Most studies are focused on the behaviour of available strength and stiffness of moment connections through the elastic zone. Limited researches are conducted on the behaviour of slant end-plate connections subjected to elevated temperature. The main objective of the present research is to generate an analytical model in order to reveal the thermal behavioural of the slanting connection with nonsymmetric gravity load.

## 2. The Reaction of Axially Restrained Steel Beams under Elevated Temperature

The reaction and failure of the beam subjected to temperature increase almost depends on the section area, boundary conditions, span, properties of material, and the amount of elevated temperature. Thermal expansions of the materials are a vital behaviour that should be considered through the analysing of the heated beam. The steel beam is a structural member that is expected to carry gravity loads. For the beam which is completely or partially restrained axially, the expansion due to elevated temperature can cause a huge axial force in the extent of confined beam. This force can be a demerit for the structural performance. The axial force in a restrained heated beam is given by (1) to (2) as follows:

(1) $$\begin{array}{c} \Delta L=\left(\frac{PL}{AE}\right),\\ \Delta L=\alpha L\Delta T. \end{array}$$

From (1), the axial load due to increase in temperature can be obtained as given in (2). From (2), a heated steel beam with a fully axially restrained supports must have enough strength against the additional axial force. In designing a nonheated beam, increase in the section area has direct influence on the amount of member's strength. However, in a heated beam, increase in the section area only cannot increase strength of beam (i.e., beam member) against the axial load. In such condition, axial force should be satisfied by two equations where the first equation presents the stress in pure axial load and the second equation shows the axial load due to elevated temperature as follows:

(2) $$\begin{array}{c} P_{t}=\alpha AE\Delta T. \end{array}$$

## 3. Vertical and Slant End-Plate Connection Characteristics in Beam Subjected to Temperature Increase

### 3.1. Connections Characteristics

**Figure 1:**
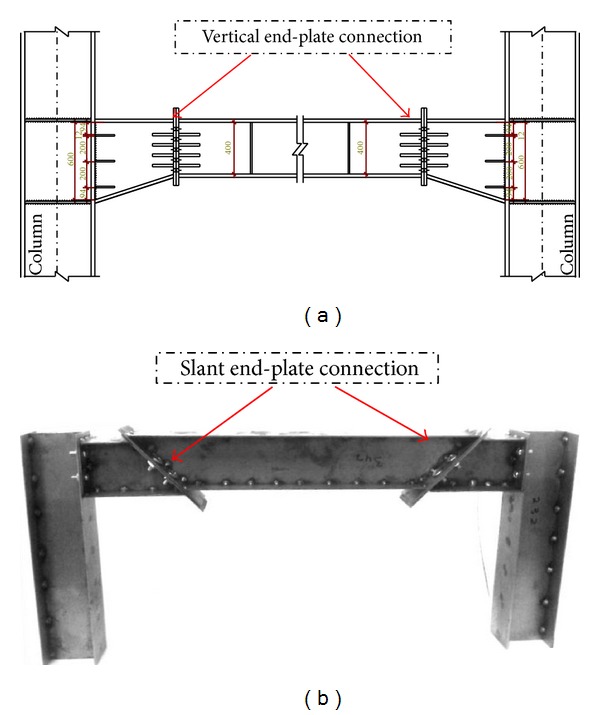
Typical beam with (a) vertical and (b) slant bolted end-plate connection.

The end-plate steel connections consist of a plate which is welded at end of the beam. The plates are bolted to the flange of columns or supported end-plate on site. The vertical end-plate connections are commonly used in the industrial structures, and the residential towers with the knee connections. The popularity of bolted end plate connection is largely due to its simplicity in fabrication and installation. Although, the slant end-plate connections are similar to the vertical models, they are different on the end-plate connection angle. A schematic view of vertical and slant end-plate connections are shown in Figure 1.

**Figure 2:**
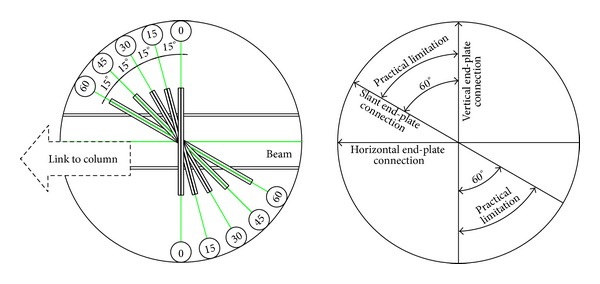
Practical angles in slant end-plate connection.

The connections of two slant plates (i.e., the joint surface) should be taken practically. For example, during the fabrication of slanted end-plate connections of beams, the angle of the slant was limited to about 60 degrees. A higher angle is not practical since the crossing bolts into holes in top and bottom of the end-plate cannot be tightened (Figure 2).

### 3.2. Crawl of Beam on Connection Surface due to Increase in Temperature

The beam with the vertical end-plate connections and fixed supports tends to have an expansion when it is subjected to temperature increase. As showed in (2), an extensive axial force in beams can be produced when the supports are not allowed moving horizontally. However, the slant end-plate connection damps the axial force by allowing the end of beam to crawl on connection surface. Such sliding is due to elongation of the original beam member.

**Figure 3:**
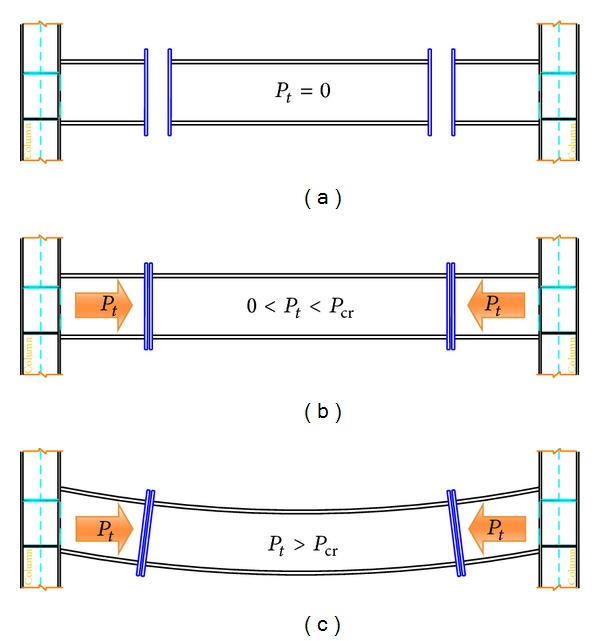
Beam with vertical bolted end-plate connection subjected to temperature increase. (a) Stage 1: beam connections before increasing the temperature. (b) Stage 2: beam connection after increase in temperature “contact two plates together.” (c) Stage 3: beam connection after increase in temperature “buckling and decrease Young's modules.”

**Figure 4:**
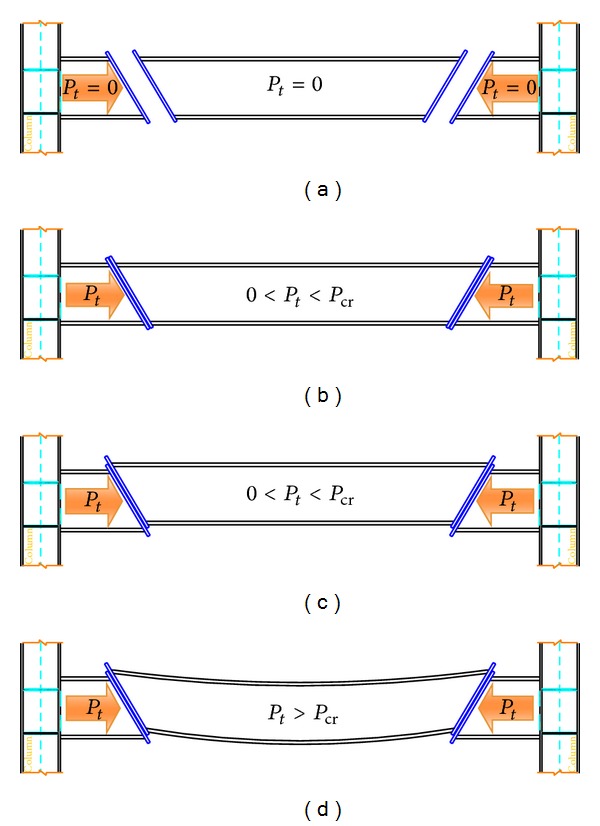
Beam with slant bolted end-plate connection subjected to temperature increase. (a) Stage 1: beam behaviour before increase in temperature. (b) Stage 2: beam behaviour after increase in temperature “two plates are in contact.” (c) Stage 3: Beam behaviour after increase in temperature “two plates contact together and in movement.” (d) Stage 4: Beam behaviour after increase in temperature “buckling and decrease in Young's modulus.”

Hypotheses about the stages of the performance and the reaction of end-plate connections due to increase in temperature are shown in Figures 3 and 4. In the conventional end-plate connection (vertical), after an increase in temperature, the beam tends to buckle due to increase in axial load. Vertical end-plate connection does not allow beam to have expansion, as shown in Figure 3. On the other hand, in the slant connection, by increase in the temperature, the support's reactions produced an axial force. The generated forces are dissipated by upward sliding on the slant surface (Figure 4).

The purpose of Figures 3 and 4 is to show that movement tolerance at the surface of end-plate connection can absorb part of the movement in the end of the beam due to elongation. Although, in vertical end-plate connection, there is small vertically movement tolerance between the surfaces, it is unable to absorb the expansion of beam horizontally as the direction of expansion is perpendicular to direction of moving surface. In the slant end-plate connection, there is a sliding surface that provides slanting tolerance where it can absorb the expansion at two ends of the beam by crawling on their slanting planes since the direction of horizontal expansion can be propelled to the slanting plane of connection.

## 4. Analytical Modelling

**Figure 5:**
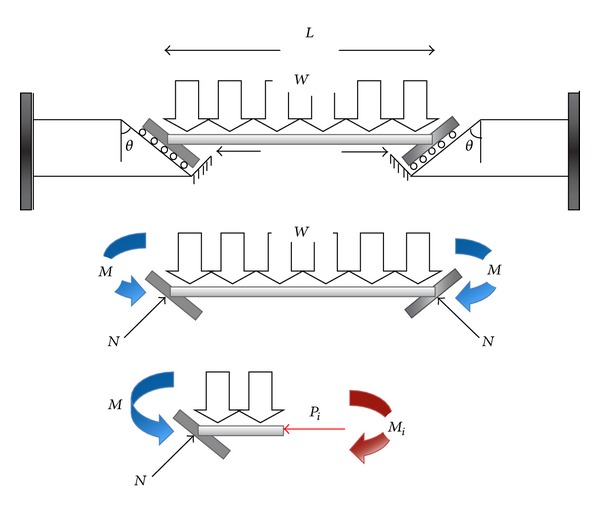
Simplification model of beam movement with slant end-plate connection due to increase in temperature and symmetric gravity load (frictionless support).

In order to simplify the calculation in two-dimensional (2D) model, joints in the end-plate connections are assumed to be rigid. The supports on these rigid cantilever beams are evaluated in three different cases, (i) first roller support, (ii) secondly friction support, and (iii) thirdly friction bolted support. For 2D simulation of the free motion of bolts in the holes of end-plate (i.e., the movement only in the allowable hole gap) it is considered that the lower part of slope line is closed and top of slope line is free (Figure 5). In the frictionless support model (Figure 5), the supports on the slant plane are assumed to be roller type. It is noteworthy that the beam should be in static equilibrium before thermal effect. As the beam is subjected to uniform symmetric gravity load, the consequent of bending moment, *M*, in the two ends of beam is zero. In static equilibrium, the uniform gravity load, *W*, causes the roller supports to move downward. However, the initial axial force into the beam, *P*_*i*_, resists against downward sliding to set up static equilibrium. In this case, if the elevated temperature is induced on the beam, it tends to move upward without any frictional resistant force from the support reaction (Figure 5). Equation (3) shows relations between uniform gravity load, *W*, and slope of connection as follows

(3) $$\begin{array}{c} \sum F_{y}=0⟶N=\frac{WL}{2\sin\theta },\\ \sum F_{x}=0⟶P_{i}=\frac{WL}{2}\text{cot}\;\theta \;\left(\text{frictionless}\right). \end{array}$$

### 4.1. The Beam with Slant End-Plate Connection Subjected to Nonsymmetric Gravity Load and Uniform Temperature Increase

In the most structures, the applied gravity load is not usually symmetric and uniform (i.e., wall load in mid span of the beam). Thus, it is necessary to consider applying nonsymmetric gravity load on the beam. In nonsymmetric gravity load case, for logical comparison, in all of the three mentioned cases, the amount of nonsymmetric gravity load is assumed to be equal to *Q*(*Q* = *WL*).

**Figure 6:**
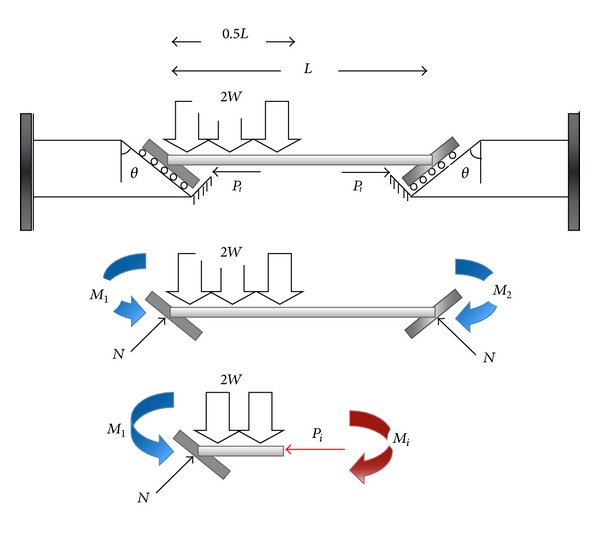
Simplification model of beam movement with nonsymmetric gravity load due to increase in temperature (frictionless support).

In the first case study, the roller supports are used on the slant plane (Figure 6). The nonsymmetric gravity load, 2*W*, make left side support to move downward, but initial axial load in the beam, *P*_*i*_, resists against movement after equilibrium in supports. Therefore, after an increase in temperature, the beam tends to move upward to damp beam elongation. Equation (4) shows relations between nonsymmetric gravity load and slope of connection.

**Figure 7:**
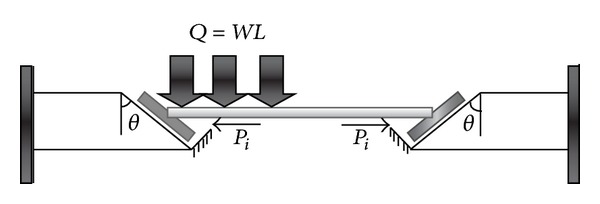
Simplification model of beam movement with nonsymmetric gravity load due to increase in temperature (friction support).

**Figure 8:**
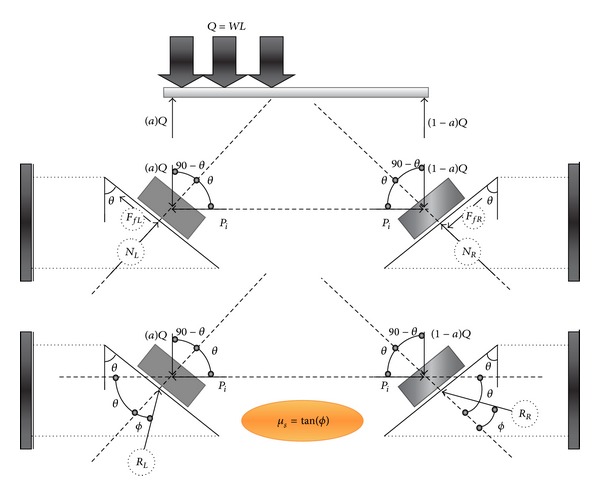
Free body diagram of beam with nonsymmetric gravity load before thermal effect (friction support).

Figure 7 presented a schematic view of the second case study. It is assumed that the reaction of supports depends on the friction factor between two faces of joint planes.  Figure 8 also illustrated the nonsymmetric gravity load, *Q*, that causes the left side support to slide downward. This also makes the support in right side to move upward (Figure 8). It should be mentioned that there are two forces that resist against movement, (i) initial axial load and (ii) friction force. The friction force resists against downward movement in the left support and upward movement in right support. Hence, the amount of reaction force in left and right sides is not the same. So, two cases can occur in equilibrium before and after sliding was started as follows:

(4) $$\begin{array}{c} \sum F_{x}=0,\;\;\sum F_{y}=0⟶N=\frac{WL}{2\sin\theta },\\ \sum F_{x}=0⟶P_{i}=\frac{WL}{2}\text{cot}\;\theta \;\text{Frictionless}. \end{array}$$

The reaction of supports can be obtained from (5), and (7). Also, the amount of initial axial load, *P*_*i*_, is calculated by (6). The Equations (5)–(7) can be derived only before the sliding started and without any thermal effect. After the slide occurred at the two ends of the beam, the reaction of supports can be derived from (9) and (10). Besides, the amount of initial axial load, *P*_*i*_, is obtained from (11). Both of cases are subjected to nonsymmetric gravity load only.

Equilibrium before sliding:

(5) $$\begin{array}{c} N_{L}=\left(aQ\right)\sin\theta +P_{i}\cos\theta , \end{array}$$

 
*F*_*fL*_ = *N*_*L*_ *μ*_*s*_,
 ∑*F*_inclined  line  Left_ = 0,
  (6) $$\begin{array}{c} P_{i}=\frac{\left(aWL\right)\left(\cos\theta -\mu_{s}\sin\theta \right)}{\sin\theta +\mu_{s}\cos\theta }=\left(aWL\right)\text{cot}\left(\theta +\phi \right), \end{array}$$
  (7) $$\begin{array}{c} N_{R}=\left(1-a\right)Q\sin\theta +P_{i}\cos\theta , \end{array}$$
 
*F*_*fR*_ = *N*_*R*_ *μ*_*s*_,
 ∑*F*_inclined  line  Right_ = 0,
 
*P*_*i*_sin*θ* − (1 − *a*)*WL*cos⁡*θ* ≤ *F*_*fR*_, equilibrium condition before sliding,
 
*P*_*i*_ = (*a* *WL*)cot(*θ* + *ϕ*) ≤ (1 − *a*)*WL*cot(*θ* − *ϕ*),
 
*a*/(1 − *a*) ≤ cot (*θ* − *ϕ*)/cot (*θ* + *ϕ*)
  (8) a≤cot⁡(θ−ϕ)cot(θ+ϕ)+cot⁡(θ−ϕ),equilibrium condition before sliding.

Equilibrium after sliding:

 ∑*F*_*x*_ = 0 → *R*_*L*_cos⁡(*θ* + *ϕ*) − *R*_*R*_cos⁡(*θ* − *ϕ*) = 0,
 ∑*F*_*y*_ = 0 → *R*_*L*_sin(*θ* + *ϕ*) + *R*_*R*_sin(*θ* − *ϕ*) = *WL*,
  (9) $$\begin{array}{c} R_{L}=\frac{WL\cos\left(\theta -\phi \right)}{\sin2\theta }, \end{array}$$
  (10) $$\begin{array}{c} R_{R}=\frac{WL\cos\left(\theta +\phi \right)}{\sin2\theta }, \end{array}$$
  (11) $$\begin{array}{c} \sum F_{x}=0⟶P_{i}=\frac{WL\left(\cos^{2}\theta -\text{si}{\text{n}}^{2}\phi \right)}{\sin2\theta }. \end{array}$$

**Figure 9:**
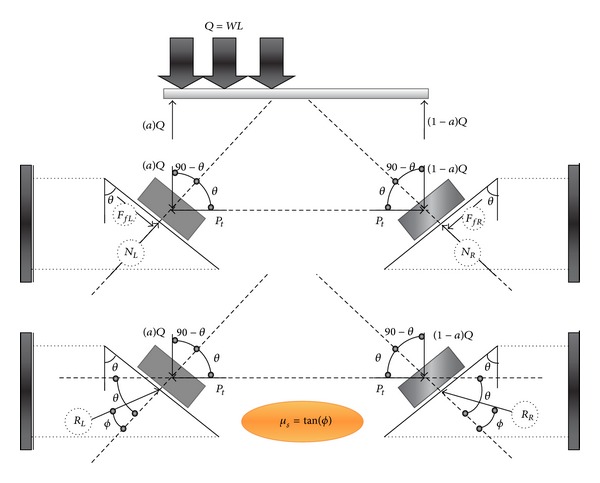
Free body diagram of beam with nonsymmetric gravity load after increase in temperature (friction support).

After increase in temperature, the beam tends to move upward on both of supports to damp extra axial load due to elongation (Figure 9). Therefore, the reaction of left side support, *R*_*L*_, will change friction vector to resist against upward movement. On the other hand, the vector's direction of reaction at the right support is still similar to before thermal effect but it resists against upward movement due to elevated temperature and gravity load. Based on the free body diagram (Figure 9), and in the second case study (after elevated temperature), the reaction of the both left and right supports, and initial axial force for the beam can be calculated from (12) and (13), respectively, as follows:

(12) $$\begin{array}{c} \sum F_{y}=0⟶R_{L}=R_{R}=\frac{WL}{2\sin\left(\theta -\phi \right)}, \end{array}$$

(13) $$\begin{array}{c} \sum F_{x}=0⟶P_{t\;\max}=\frac{WL}{2}\text{cot}\left(\theta -\phi \right)\;\text{friction}\;\text{form}. \end{array}$$

From the substitution of (13) into (2) (in the elastic zone) the movement elevated temperature (Δ*T*_*m*_) can be obtained as given in the following:

(14) $$\begin{array}{c} \Delta T_{m}=\frac{WL}{2AE\alpha }\text{cot}\left(\theta -\phi \right)\;\text{friction}\;\text{form}. \end{array}$$

**Figure 10:**
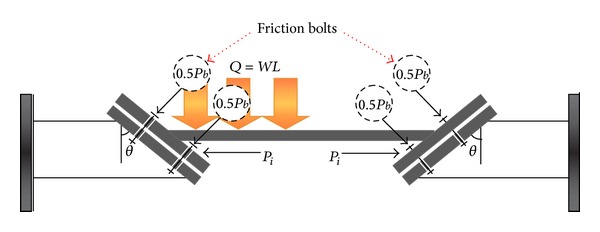
Simplification model of beam movement with nonsymmetric gravity load due to increase in temperature (friction bolted support).

**Figure 11:**
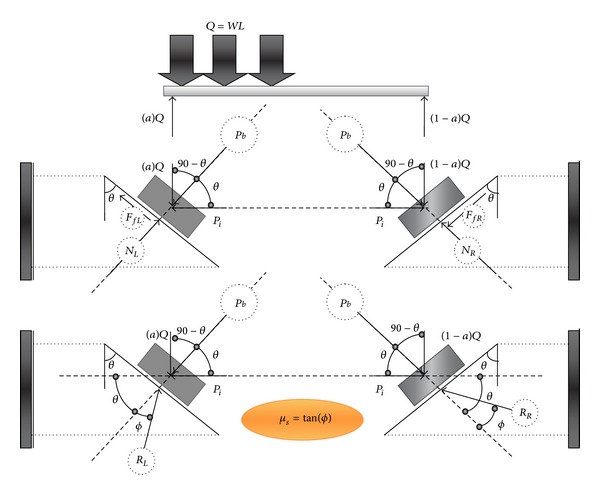
Free body diagram of beam with nonsymmetric gravity load before thermal effect (friction bolted support).

In the third case study, the reaction of supports depends on the friction factor, and friction bolts among two faces of joint place (Figure 10). In the friction bolt case, the nonsymmetric gravity load, *Q*, makes the left support to slide downward and makes right support to move upward (Figure 11). The amount of friction force in this case is greater than when normal bolts were used (case two). Both of the normal tightening force and friction force increase during the bolts fastening. Therefore, in friction bolted connection, the beam needs higher axial force to move upward when it is subjected to temperature increase.

As can be seen from Figure 11, in the case of before thermal effect, the equilibrium equations can be written as two cases (i) before start sliding (15)–(18), and (ii) after start sliding (19)–(21).

Equilibrium before sliding as follows:

(15) $$\begin{array}{c} N_{L}=\left(aQ\right)\sin\theta +P_{i}\cos\theta +P_{b}, \end{array}$$

 
*F*_*fL*_ = *N*_*L*_ *μ*_*s*_,
 ∑*F*_inclined  line  Left_ = 0,
  (16) Pi=(aWL)(cos⁡θ−μssinθ)−μsPbsinθ+μscos⁡θ,=(aWL)cot(θ+ϕ)−Pbsinϕsin⁡(θ+ϕ)
  (17) $$\begin{array}{c} N_{R}=\left(1-a\right)Q\sin\theta +P_{i}\cos\theta +P_{b}, \end{array}$$
 
*F*_*fR*_ = *N*_*R*_ *μ*_*s*_,
 ∑*F*_inclined  line  Right_ = 0,
 
*P*_*i*_sin*θ* − (1 − *a*)*WL*cos⁡*θ* ≤ *F*_*fR*_, equilibrium condition before sliding,
 
*P*_*i*_ = (*a* *WL*)cot(*θ* + *ϕ*) − *P*_*b*_sin*ϕ*/sin⁡(*θ* + *ϕ*) ≤ (1 − *a*)*WL*cot(*θ* − *ϕ*) + *P*_*b*_sin*ϕ*/sin⁡(*θ* − *ϕ*),
  (18) a≤cot(θ−ϕ)cot(θ+ϕ)+cot(θ−ϕ)+Pb2WL(cos⁡⁡(θ−2ϕ)−cos⁡⁡(θ+2ϕ)sin2θ).

Equilibrium after sliding as follows:

 ∑*F*_*x*_ = 0 → *R*_*L*_cos⁡(*θ* + *ϕ*) − *R*_*R*_cos⁡(*θ* − *ϕ*) = 0,
 ∑*F*_*y*_ = 0 → *R*_*L*_sin(*θ* + *ϕ*) + *R*_*R*_sin(*θ* − *ϕ*) = *WL* + 2*P*_*b*_sin*θ*,
  (19) $$\begin{array}{c} R_{L}=\frac{\left(WL+2P_{b}\sin\theta \right)\cos\left(\theta -\phi \right)}{\sin2\theta }, \end{array}$$
  (20) $$\begin{array}{c} R_{R}=\frac{\left(WL+2P_{b}\sin\theta \right)\cos\left(\theta +\phi \right)}{\sin2\theta }, \end{array}$$
  (21) ∑Fx=0 ⟶Pi=(WL+2Pbsinθ)(cos⁡2⁡θ−sin2ϕ)sin2θ−Pbcos⁡θ.

**Figure 12:**
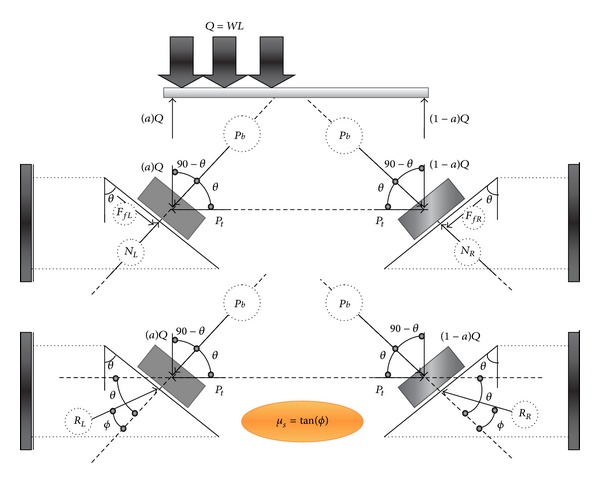
Free body diagram of beam with nonsymmetric gravity load after increase in temperature (friction bolted support).

After increase in temperature, the beam tends to move upward on the left and right supports in order to control extra axial force due to expansion of the beam. Thus, the left support's reaction, *R*_*L*_, reverses the friction vector to resist against upward movement. However, the right reaction of support keeps steady as before to be heated. The friction force resisted against upward movement due to only nonsymmetric gravity load. However, in new position, the right support's reaction increases to resist against upward movement due to both nonsymmetric gravity load and elevated temperature.  Figure 12 illustrates the process of generating (22) and (23).

(22) $$\begin{array}{c} \sum F_{y}=0⟶R_{L}=R_{R}=\frac{WL+2P_{b}\sin\theta }{2\sin\left(\theta -\phi \right)}, \end{array}$$

(23) $$\begin{array}{c} \sum F_{x}=0⟶P_{t\;\max}=\frac{WL+2P_{b}\sin\theta }{2}\text{cot}\left(\theta -\phi \right)-P_{b}\cos\theta . \end{array}$$

By merging (2) and (23), the movement elevated temperature in this case can be obtained as follows:

(24) $$\begin{array}{c} \Delta T_{m}=\frac{1}{\alpha AE}\left(\frac{WL+2P_{b}\sin\theta }{2}\text{cot}\left(\theta -\phi \right)-P_{b}\cos\theta \right). \end{array}$$

### 4.2. Critical Axial Load in the Beam-Column

**Figure 13:**
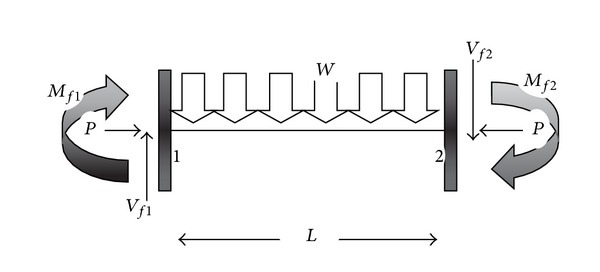
Beam column due to axial load [9].

The free body diagram of the beam-column with the uniform symmetric gravity load, *W*, is presented in Figure 13. It is assumed that the right and left supports are fixed at both ends of the beam. Based on the equilibrium equations and the common relations for the beam-columns, the critical load, *P*_cr_, and, *ν*_c_ can be written [9] as follows:

(25) $$\begin{array}{c} EI\left(\frac{d^{4}\nu }{dx^{4}}\right)+P\left(\frac{d^{4}\nu }{dy^{4}}\right)=W\left(x\right). \end{array}$$

After solving (25), the critical load of the beam column from Euler formulation in buckling concept can be obtained as follows:

(26) $$\begin{array}{c} P_{\text{cr}}=\left(\frac{\pi^{2}EI}{k^{2}L^{2}}\right). \end{array}$$

**Figure 14:**
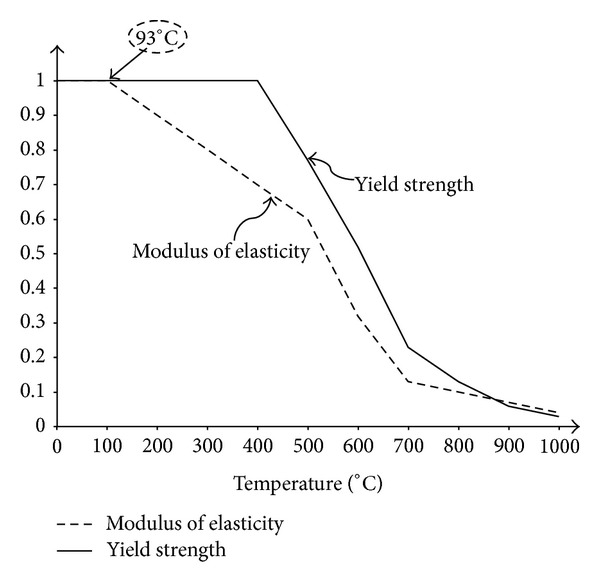
Reduction in yield strength and modulus of Elasticity of steel with temperature [10].

The elastic zone that is mentioned through this study is shown in Figure 14. The curve of this figure shows that reference temperature plus increase in the temperature, Δ*T* should be less than 93°C in order to be in the elastic zone. By substituting (26) in (2), the critical elevated temperature of beam buckling can be obtained as given in (27).

(27) $$\begin{array}{c} \Delta T_{\text{cr}}=\frac{1}{\alpha }\left(\frac{\pi r}{kL}\right){}^{2}=\frac{1}{\alpha }\left(\frac{\pi }{\lambda }\right){}^{2}, \end{array}$$

where *λ* = *kL*/*r*.

**Figure 15:**
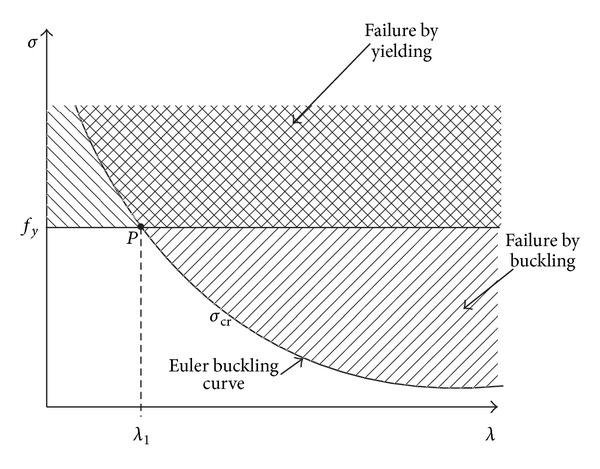
The relationship between buckling strength and slenderness ratio depends on the support conditions at the column ends [11].

When the beam-columns are subjected to both axial force and bending moment, it may yield before any buckling occurred. However, this is mainly dependent on the slender ratio, *λ*, and section properties (Figure 15). The allowable axial yielding load, *P*_*y*_ can be obtained from (28). This equation is resulted from the substitution of allowable yielding stress due to axial load, *F*_*a*_, and bending moment, *F*_*b*_. In addition, in (28), the applied stresses due to axial load, *f*_*a*_, and bending moment, *f*_*b*_, are varied where it changes by the external loads.

(28) $$\begin{array}{c} \frac{f_{a}}{F_{a}}+\frac{f_{b}}{F_{b}}\leq 1. \end{array}$$

As stated in (2) and (26), *P*_cr_ and *P*_*t*_ can be obtained, respectively. Axial load that is produced by increasing in the temperature, *P*_*t*_, is equal to, *P*_cr_, when the amount of increase in temperature plus ambient temperature is less than 93°C. The modules of elasticity component in steel structure are linear and elastic in this temperature zone. Critical elevated temperature can be obtained from (27). It should be mentioned that an increase in slenderness ratio of beam-column will reduce the ability of strength against elevated temperature (27). However, when we exceed the critical temperature, a reduction factor should be applied to the amount of elasticity modules since the material behaviours go to the inelastic zone.

In conventional end-plate connections (vertical), after an increase in temperature, the beam tends to yield or buckle due to increase in axial force. This is due to the face that vertical end-plate connections do not allow the beam to have an elongation. However, the inclined surfaces at two ends of beam in the slant end-plate connection allow the beam to damp axial force and elongation with linear crawling on slant surface. From (2), (26), and (28), it can be found that if *P*_cr_ and *P*_*y*_ are to be less than *P*_*t* max⁡_, then the beam will yield or buckle. On the other hand, if *P*_cr_ and *P*_*y*_ resulted to be higher than *P*_*t* max⁡_, then the beam will crawl upward before buckling and additional axial force due to elongation will be damped.

## 5. Illustration

The structural model described in the previous section has been used to analyse a steel beam section at elevated temperature, in which an IPE 300 beam is connected at its ends to slant fixed end-plate supports (Figure 7). For the beam: cross section area (*A*) = 5380 mm^2^, modules of elasticity (*E*) = 210 kN/mm^2^, ambient temperature (*T*0) = 20°C, elevated temperature (Δ*T*) = 50°C, length of beam column (*L*) = 6000 mm, coefficient of thermal expansion (*α*)= 1.5 × 10^−5^ 1/°C, and linear nonsymmetric gravity load for half length of span (*W*) = 40 kN/m (*Q* = 40 × 3 = 120 kN). Axial applied force on friction bolts (*P*_*b*_) is equal to 50 kN (total force). The slope of slant end-plate connection (*θ*) and friction coefficient factor (*μs* = tan*φ*) is varied.

**Figure 16:**
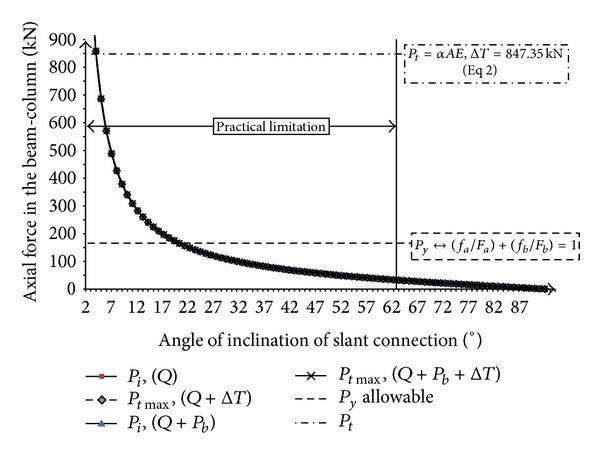
Relation between axial force in the beam with slope of slant connection (*θ*°) due to nonsymmetric gravity load and elevated temperature (friction factor, *μs* = tan*φ* = 0) (Δ*T*) = 50°C.

**Figure 17:**
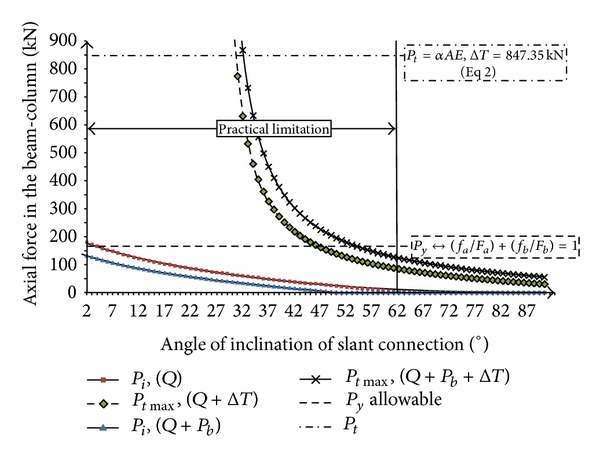
Relation between axial force in the beam with slope of slant connection (*θ*°) due to nonsymmetric gravity load and elevated temperature (friction factor, *μ*_*s*_ = tan*φ* = 0.5) (Δ*T*) = 50°C.

**Figure 18:**
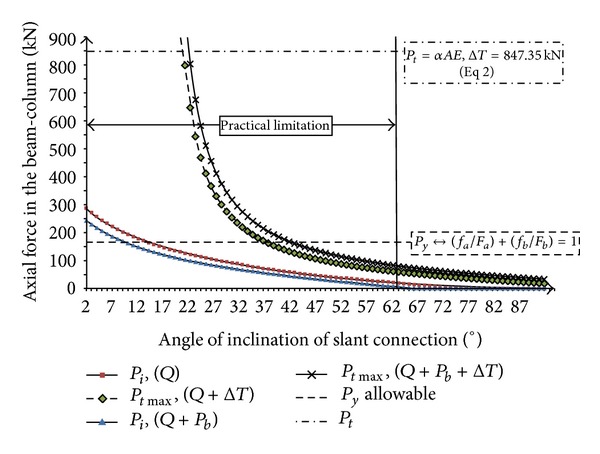
Relation between axial force in the beam with slope of slant connection (*θ*°) due to nonsymmetric gravity load and elevated temperature (friction factor, *μ*_*s*_ = tan*φ* = 0.3) (Δ*T*) = 50°C.

**Figure 19:**
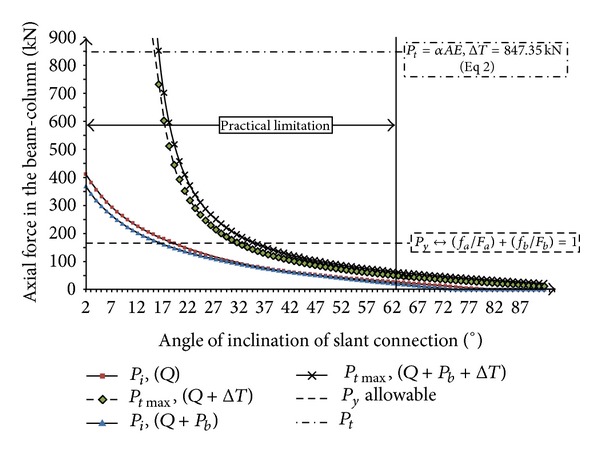
Relation between axial force in the beam with slope of slant connection (*θ*°) due to nonsymmetric gravity load and elevated temperature (friction factor, *μ*_*s*_ = tan*φ* = 0.2) (Δ*T*) = 50°C.

Variation of induced axial force in the beam with angle of slant connection, *θ*, due to nonsymmetric gravity load and elevated temperature which are shown in Figures 16 to 19. As the supports are assumed to be frictionless (*μs* = 0), the resulted axial forces are similar with both nonsymmetric gravity load (before elevated temperature) and increase in temperature (after elevated temperature) (Figure 16). Because after substitution of friction factor (*μs* = 0) in (6), (11), (13), and also (16), (21), and (23), the same results are obtained. It can be concluded that by increasing the angle of connection from vertical to slant position, the axial force reaction will be decreased.

On the other hand, when there is friction between two faces of connection plate, the obtained results of initial axial force due to elevated temperature and gravity load are different.  Figure 17 presented the relation between axial force in the beam with slope of slant connection (*θ*°) due to nonsymmetric gravity load and elevated temperature (friction factor, *μs* = tan *φ* = 0.5) (Δ*T*) = 50°C. The curve of axial force due to only nonsymmetric gravity load (*P*_*i*_, *Q*) starts from 187 kN (angle = 2°), in vertical end-plate position and it goes to zero by increasing the angle of slant connection. It should be mentioned that when the angle of slant connection is equal to 64°, the axial force is reduced to zero (Figure 17). When the friction bolts used (*P*_*i*_, *Q* + *P*_*b*_), the axial force starts from 138 kN in the vertical position and it goes to zero where the angle of slant connection is equal to 50°. Therefore, it can be concluded that the induced axial force for beam in case of before elevated temperature can be decreased by friction bolts (Figure 17).

In case of after elevated temperature, the minimum axial force that is required to begin crawling in the beam (*P*_*i*_, *Q* + Δ*T*) starts from infinity where the angle of end-plate is in its vertical form. The axial force decreases by increasing in the angle of slant connection and it vanishes when the angle of slant connection goes to 90°. As stated earlier, the practical angle of slant end-plate connection is limited from 0° to 60°. Thereby, the minimum requirement axial force of the beam to begin crawling (*P*_*i*_, *Q* + Δ*T*), is 90.87 kN where the angle of slanting is equal to 60° (Figure 17). Furthermore, when friction bolt is used, the minimum requirement of axial force of beam to start the movement (*P*_*i*_, *Q* + *P*_*b*_ + Δ*T*) is higher than the case of normal bolted connection (*P*_*i*_, *Q* + Δ*T*). Thus, it can be concluded that if the friction bolts are used instead of normal bolts, we need higher axial force and elevated temperature to make two ends of the beam aiming for upward crawling on the slant plane.

Figure 18 shows the relation between axial force in the beam with slope of slant connection (*θ*°) due to nonsymmetric gravity load and elevated temperature having the effective friction factor equal to 0.3 (*μs* = tan *φ* = 0.3). The axial forces before thermal effect start from 287 kN (angle = 2°) when the beam is subjected to only gravity load (*P*_*i*_, *Q*) and 242 kN when the beam is subjected to both of the gravity load and effect of friction bolts (*P*_*i*_, *Q* + *P*_*b*_). In both cases of *P*_*i*_, *Q* and *P*_*i*_, *Q* + *P*_*b*_, the axial force goes to zero at the angle of 73° and 65°, respectively. It indicates that the influence of friction bolts in decreasing the induced axial force before the increase in temperature is similar to the previous case (*μs* = 0.5). Also, by comparison of Figures 17 and 18, it can be resulted that by decreasing the friction factor, *μs*, from 0.5 to 0.3, the induced axial force will rise. After increase in temperature, the minimum axial force in the beam for starting of crawling for both of the cases, *P*_*i*_, *Q* + Δ*T* and *P*_*i*_, *Q* + *P*_*b*_ + Δ*T* at the angle of end-plate connection of 60°, are 63.67 kN and 84.62 kN, respectively. Hence, in comparing to the previous case (*μ*_*s*_ = 0.5), the beam starts to crawl on the slant plane with a lower initial axial force.  Figure 19 shows the relation between axial force in the beam with the slope of slant connection (*θ*°) due to nonsymmetric gravity load and elevated temperature with effective friction factor, *μ*_*s*_ = 0.2.

The friction bolts can be useful to decrease the induced axial force of beam before any thermal effect. However, it can also be harmful if we ignore the damping behaviour of bolted slant end-plate connection when it is subjected to temperature increase. It can be harmful because by increasing in the normal force of friction bolt, the minimum requirement axial force in beam for starting of movement (*P*_*i*_, *Q* + *P*_*b*_ + Δ*T*) will increase. Noteworthy, it is possible that before any crawling at two ends of the beam on slant connection surface, the beam will start to yield or buckle.

**Figure 20:**
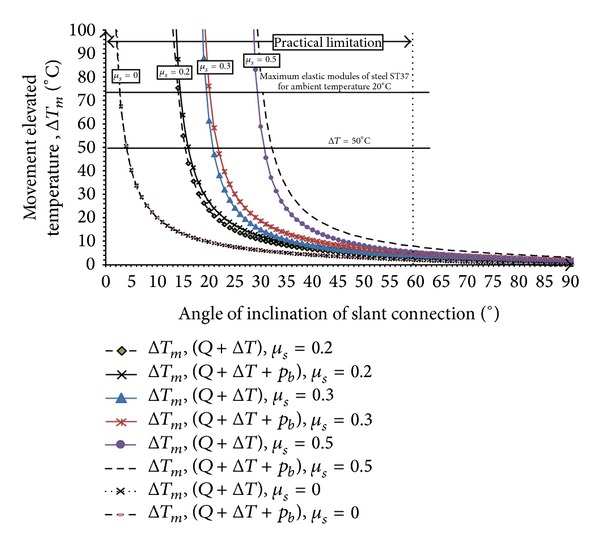
Relation between movement elevated temperature, Δ*T*_*m*_, and slope of slant connection (*θ*°) due to nonsymmetric gravity load.

Figure 20 shows the variation of minimum increase in temperature, Δ*T*_*m*_, in the beam with the angle of slant connection, *θ*, under nonsymmetric gravity load and normal force of friction bolt, *P*_*b*_. The amount of elevated temperature depends on nonsymmetric gravity load and the amount of applied normal force by friction bolts. After substitution of zero friction factor, *μs* = 0, in (14) and (24), for nonsymmetric gravity load case, the same results for movement elevated temperature, Δ*T*_*m*_, can be obtained. Therefore, it can be found that by an increase in angle of connection from vertical to slant position, the amount of movement elevated temperature, Δ*T*_*m*_, will decrease. By approaching to conventional connection (vertical), the required elevated temperature for the movement goes to infinity. As shown in Figure 14, the behaviour of steel member in the elastic field subjected to elevated temperature is limited to 93°C, so the maximum elevated temperature in this illustration is equal to 73°C.

The amount of movement elevated temperature, Δ*T*_*m*_, alters with considering the friction force at two faces of the connections. However, it depends on nonsymmetric gravity load and normal force of friction bolt (Figure 20). In addition, if the angle of end-plate, *θ*, is equal to friction angle, *φ*, then the elevated movement temperature, Δ*T*_*m*_, in the beam goes to infinity. Besides, when the slant connections' angle, (*θ*), is equal to 90°, the slope of end-plate (tan*θ*) goes to infinity. As a result, the amount of movement elevated temperature, Δ*T*_*m*_, goes to zero.

It can be concluded that if the end-plate angle is approached to a vertical form, the required elevated temperature for primary movement will be approached to the infinity. On the other hand, if the end-plate angle is approached to the horizontal position, the amount of required elevated temperature for primary movement will near to zero.

From Figure 20, it can be concluded that increasing in the friction factor from 0.0 to 0.5, leads to increase in the amount of minimum elevated temperature for movement, Δ*T*_*m*_. It means that the friction force resists against upward crawling at the ends of the beam. It needs higher elevated temperature to start moving. In addition, the normal force of friction bolts can decrease the sliding friction force against upward movement of the beam.

## 6. Conclusion

This paper reports the development of linear analytical modelling of beam with bolted slant end-plate connection at both ends subjected to uniform temperature increase and nonsymmetric gravity load. The results of analytical analyses and the illustration showed that the thermal expansion of the beam may cause huge axial force in the element. It can also be the primary cause to decrease in strength of the beam and increase in deflection against gravity loads. The proposed slant end-plate connection could successfully damp huge thermal induced axial force by friction sliding and movement on the slope surface of end-plate. The results also proved that the type of applied gravity load (i.e., nonsymmetric instead of symmetric gravity load), for a particular amount of gravity load, does not induce any change to the thermal reaction of supports at slant end-plate connections. In addition, the amount of initial axial force, *P*_*t*_, in the beam is similar between nonsymmetric and symmetric gravity loads when it is subjected to temperature increase. However, the amount of initial axial load, *P*_*i*_, when the beam is subjected to only nonsymmetric gravity load (before elevated temperature) is greater than the symmetric case of gravity load. It depends on the reaction of supports due to nonsymmetric gravity load.

The minimum required elevated temperature for crawling at the ends of the beam, Δ*T*_*m*_, will increase where the amount of friction factor changes from 0 to 0.5 (with increase of the friction resistance). The friction force resists against upward crawling of the end of beam. In the case where friction bolts are used, the friction bolts can decrease the sliding friction force against upward movement of the beam. It is possible to optimize the design that has enough ability to absorb the huge axial force by friction and movement damping system before any yielding and buckling in the beam.

## Nomenclature

*E*: Young's modulus
*I*: Moment of inertia
*L*: Length of the beam column
*P*_cr_: Critical compressive axial load
*P*_*t*_: Axial load due to increase in temperature
*ν*: Deflection function
*P*: Compression axial load
*P*_*E*_: Critical compressive load of an elastic pin-ended beam column
*P*_*m*_: Movement axial load due to increase in temperature
*W*: Distributed load (gravity load)
*α*: Coefficient of thermal expansion
Δ*T*: Additional temperature
*A*: Cross section of beam column
Δ*T*_cr_: Critical additional temperature
Δ*T*_*m*_: Movement temperature
*λ*: Slender ratio
*μ*_*s*_: Friction factor
*r*: Radius of gyration
*M*: Bending moment
*a*: Vertical reaction factor of support due to nonsymmetric gravity load.
